# Adverse health outcomes among migrant workers and transnational families in the Asia–Pacific: a systematic review and meta-analysis

**DOI:** 10.1016/j.lanwpc.2025.101720

**Published:** 2025-10-28

**Authors:** Rosita Chia-Yin Lin, Karen Lau, Kathryn Mackey, Natasha Roya Matthews, Maushmi Selvamani, Morais Beatriz, Bouaddi Oumnia, Chaelin Kim, Azusa Iwamoto, Masami Fujita, Ursula Trummer, Tran Ngoc Dang, Alena Kamenshchikova, Cathy Zimmerman, Sally Hargreaves

**Affiliations:** a The Migrant Health Research Group and the Consortium for Migrant Worker Health, The School of Health and Medical Sciences, Institute for Infection and Immunity, City St George's, University of London, London, UK; b Department of Global Health & Development, London School of Hygiene & Tropical Medicine, London, UK; c Department of Infectious Disease Epidemiology and Dynamic, London School of Hygiene & Tropical Medicine, London, UK; d Emergency Department Clinical Research Group, St George's University Hospitals NHS Foundation Trust, London, UK; e Schizophrenia Research Foundation India (SCARF Hospital), Chennai, India; f Mohammed VI International School of Public Health, Mohammed VI University of Sciences and Health, Casablanca & Mohammed VI Center for Research and Innovation (CM6RI), Rabat, Morocco; g Bureau of Global Health Cooperation, Japan Institute for Health Security, Tokyo, Japan; h Centre for Health and Migration, Vienna, Austria; i Faculty of Public Health, University of Medicine and Pharmacy at Ho Chi Minh City, Viet Nam; j Department of Health, Ethics and Society, Maastricht University, Maastricht, Netherlands; k Department of Social Medicine, Maastricht University, Maastricht, Netherlands; l Department of Medical Microbiology, Infectious Diseases and Infection Prevention, Maastricht University, Maastricht, Netherlands

**Keywords:** Migrant worker, Transnational family, Left-behind children, Asia–Pacific, Health outcome

## Abstract

**Background:**

Labour migration is a growing global phenomenon, with migrant workers known to be more likely to experience exploitative and precarious work, impacting their health. Despite hosting over 27 million international migrant workers, the Asia–Pacific region remains underrepresented in global health evidence, limiting the development of targeted, region-specific health interventions. This study aims to investigate the health status of migrant workers and their transnational families in the Asia–Pacific region.

**Methods:**

We conducted a systematic review and meta-analysis (PROSPERO: CRD42024528561) of primary studies published between Jan 1, 2013, and April 1, 2024. We searched MEDLINE, Embase, PsycINFO, and Ovid Global Health for studies reporting work-related morbidity and mortality among international migrant workers and their transnational families in the Asia–Pacific region. A narrative synthesis identified occupational risks; a meta-analysis estimated pooled prevalence of morbidity among migrant workers and relative risks compared to local workers.

**Findings:**

Of 2877 studies identified, 54 met inclusion criteria, including 38 eligible for meta-analysis, encompassing 86,620 individuals across 17 Asia–Pacific countries. Among migrant workers (n = 64,172), 88.4% experienced occupational injuries and illnesses including work-related injuries, pesticide poisoning symptoms and respiratory diseases (n = 45,661), with 75% of migrant workers in this large dataset working in high-risk sectors such as manufacturing, construction, and services (including drivers and restaurant workers). 3.8% reported symptoms of mental health disorders such as anxiety and depression (n = 1975), and 3.8% had musculoskeletal disorders (n = 1973). The pooled prevalence of at least one work-related morbidity was 37% (95% CI: 27–47; *I*^*2*^ = 99.0%), with a pooled relative risk of 1.29 (95% CI: 1.10–1.52; *I*^*2*^ = 47.4%) compared with local workers. Among transnational families left behind in the migrant worker's country of origin (n = 22,448), 50.1% reported mental health issues (n = 1520), and 31.4% experienced undernutrition (n = 954). Key contributing factors to poor health outcomes of migrant workers included long working hours, workplace hazards, precarious working conditions, and healthcare access barriers.

**Interpretation:**

Migrant workers in the Asia–Pacific face substantial risks of a wide range of occupational injuries and illnesses. Although some studies support the “healthy migrant effect,” this advantage clearly diminishes over time due to cumulative exposure to occupational and structural stressors. Strengthening occupational safety, regulating working hours, and improving healthcare access, are urgent priorities for countries hosting large migrant worker populations and employers of migrant workers.

**Funding:**

Ministry of Education, Taiwan; 10.13039/100010269Wellcome Trust (318501/Z/24/Z and 335954/Z/25/Z); 10.13039/501100000265UK Medical Research Council (MR/W006677/1); UK National Health Institute for Health and Care Research (NIHR209895); the 10.13039/100010434‘la Caixa' Foundation (LCF/PR/SP21/52930003).

**Research in context**

**Evidence before this study**

Literature on the health of migrant workers and their transnational families—including left-behind children—has consistently reported adverse outcomes globally. Three systematic reviews have examined occupational health among migrant workers. Hargreaves et al. reported a 47% pooled prevalence of at least one occupational morbidity among 7260 migrant workers (95% CI: 29–64). Lau et al. found a significantly higher risk of fatal injuries among migrants when compared with local workers (relative risk = 1.71; 95% CI: 1.22–2.38), while Pega et al. reported higher occupational injury rates (relative risk = 1.27; 95% CI: 1.11–1.45). Regarding transnational families, a global review by Fellmeth et al. showed left-behind children were at increased risk of depression (relative risk = 1.52; 95% CI: 1.27–1.82), anxiety (relative risk = 1.85; 95% CI: 1.36–2.53), wasting (relative risk = 1.13; 95% CI: 1.03–1.24), and stunting (relative risk = 1.12; 95% CI: 1.00–1.26) compared to non-migrant households. Antia et al. similarly reported higher levels of depression and loneliness in left-behind children.

According to updated estimates by the International Labour Organization (ILO) in 2022, the Asia–Pacific region hosts approximately 27.2 million migrant workers, accounting for 16% of the global migrant workforce. Despite growing interest in the health impacts of labour migration and the potential to leverage work as a platform for health improvement, the Asia-Pacific—one of the world's major hubs for labour migration—has not been the focus of a comprehensive analysis. Evidence on the health effects of labour migration in the region remains limited, particularly regarding the health outcomes of both migrant workers and their transnational families.

**Added value of this study**

This systematic review and meta-analysis provide the first comprehensive synthesis of health outcomes among migrant workers and their transnational families, including children, spouses and adult relatives left-behind in the countries of origin, in the Asia–Pacific region. Based on data from 86,620 individuals, we found that over 75% of migrant workers worked in high-risk sectors such as manufacturing, construction, and services. Among migrant workers, 88.4% reported occupational injuries and illnesses (n = 45,661), 3.8% experienced symptoms of mental health disorders (n = 1975), and another 3.8% reported musculoskeletal disorders (n = 1973), largely associated with precarious working conditions and limited access to healthcare. Health impacts of labour migration also extended to transnational families. Mental health issues—including suicide attempts and psychiatric diagnoses among children—were reported in 50.1% of cases (n = 1520), and 31.4% experienced undernutrition (n = 954).

**Implications of all the available evidence**

The health needs of migrant workers in the Asia–Pacific remain insufficiently addressed in both research and policy, despite their vital economic contributions. Global frameworks such as Sustainable Development Goal (SDG) 8.8 call for stronger health protections through coordinated efforts between labour-sending and -receiving countries—efforts that require robust, context-specific evidence. This gap underscores the urgent need for evidence-based strategies to improve occupational safety, regulate working hours, and ensure healthcare access. Given the cross-border impacts on transnational families, stronger coordination and integration of global frameworks into national policies are essential to protect migrant health and well-being.

## Introduction

Labour migration is a global phenomenon, with 167.7 million migrant workers worldwide in 2022–4.7% of the global labour force—reflecting a 30 million increase since 2013 (ILO).^1^ Nearly 70% reside in high-income countries, mainly in agricultural, industrial, and service sectors.^1^ Evidence shows substantial health risks among migrant workers. Hargreaves et al. reported high rates of musculoskeletal disorders (MSDs), dermatological infections, depression, and occupational injuries, largely due to precarious conditions (low wages, long hours, undocumented status).^2^ Pega et al. found higher occupational injury morbidity in migrant versus local workers (relative risk = 1.27; 95% CI: 1.11–1.45),^3^ and Lau et al. reported a higher risk of fatal workplace injuries (relative risk = 1.71; 95% CI: 1.22–2.38), despite migrants often being healthier at baseline.^4^ Labour migration also affects transnational families, with studies by Fellmeth et al. and Antia et al. showing that left-behind children face greater risks of mental health conditions and undernutrition, including being underweight, compared to peers in non-migrant households.^5^^,^^6^

Despite these known challenges, research specifically examining the health of migrant workers and their transnational families in the Asia–Pacific region remains limited, even though the region hosts approximately 27.2 million migrant workers.^1^ In the Asia–Pacific, this reality is exemplified by the Philippines, where large numbers of overseas Filipino workers leave behind children who often experience reduced parental involvement and face heightened risks of adverse health outcomes.^7^

Several international frameworks have been established to protect the health and rights of migrant workers. The World Health Organization Global Plan of Action on Workers’ Health 2008–2017 (WHO GPA) called for reducing health disparities between migrant and local workers.^8^ Within the United Nations Sustainable Development Goals (SDGs), Goal 8.8 promotes safe and secure working environments for all workers, including migrants.^9^ The Global Compact for Safe, Orderly and Regular Migration (Objective 6) and The IOM emphasise fair and ethical recruitment to ensure decent work.^10^^,^^11^ Moreover, a recent commentary has highlighted the critical need to address labour exploitation in the global workforce.^12^

Although the Asia–Pacific region accounts for 16.2% of the global migrant workforce,^1^ most research on migrant workers' health has focused on the USA and Europe. This evidence gap limits the development of effective, region-specific health policies for migrant workers. Studies from parts of Asia have reported mental health risks and limited healthcare access among migrant workers, but these findings remain fragmented, without comprehensive regional synthesis.^13^^,^^14^ Similarly, research from the Greater Mekong Subregion has highlighted substantial health risks linked to limited access to healthcare services.^15^ Despite the region's scale of labour migration, systematic reviews that consolidate evidence across Asia–Pacific countries are lacking, in contrast to those available for Europe and the USA. Evidence on the health of their transnational families left behind in countries of origin, is also sparse and has largely focused on left-behind children in China, primarily within the context of internal rather than international labour migration.^5^

To address this gap, we conducted a systematic review and meta-analysis on the health of migrant workers and their transnational families in the Asia–Pacific. We examined work-related health outcomes and quantified morbidity prevalence and relative risks to inform policies on occupational safety and healthcare access.

## Methods

### Search strategy and inclusion criteria

We systematically searched Embase, MEDLINE, Ovid Global Health, and PsychINFO for primary studies published between Jan 1, 2013, and April 1, 2024. The starting year was chosen because the International Labour Organization (ILO) provides Asia–Pacific regional estimates of international migrant workers only from 2013 onwards, allowing our review to align with the most recent period of comparable labour migration data. Studies were eligible if they reported health outcomes among international migrant workers or their transnational families within the Asia–Pacific region. No language restrictions were applied. A Boolean search strategy was developed by RCL based on previous literature,^2^^,^^4^^,^^5^ and subsequently refined by SH. The full search strategy is provided in Appendix pp 1–2. In addition, grey literature and non-English studies were manually searched using key terms in Google Scholar, Google Search, and websites of relevant international and regional organisations, including WHO, IOM and ILO.

International migrant workers were defined according to the UN Convention on the Protection of the Rights of All Migrant Workers and Members of Their Families,^16^ as individuals engaged in remunerated work in a country where they are not nationals, regardless of documentation status. Eligible studies included those reporting on international migrant workers who originated from and were employed within the Asia–Pacific region, as defined by the United Nations regional classification,^17^ with the addition of Taiwan, encompassing a total of 56 countries and territories (Appendix p 3). Transnational families were defined as households in which at least one member was employed overseas (with no geographical restriction on the country of employment), while the remaining family members resided in their country of origin within the Asia–Pacific region. This group included left-behind children, stay-behind spouses, older adults, and caregivers. We included all types of primary research designs as long as they reported empirical data. However, studies that combined migrant and local workers, or international and internal migrant households, were excluded unless data were disaggregated and reported separately. Health outcomes for international migrant workers were defined as morbidity and mortality linked to their occupation, work environment, or workplace exposures. For transnational families, health outcomes referred to morbidity and mortality associated with the migration of family members employed abroad.

Titles and abstracts of English-language primary studies were screened by four reviewers (RCL, KL, KM, NR). Grey literature and non-English studies were manually searched and screened by five reviewers (RCL, KL, MS, CK, AI). All records were independently screened by two reviewers using the web-based application Rayyan,^18^ with discrepancies resolved by consensus.

The review was conducted in accordance with the Preferred Reporting Items for Systematic Reviews and Meta-Analyses (PRISMA) guidelines.^19^ This study is registered with PROSPERO, number CRD42024528561.

### Data analysis

Full-text articles were screened and data extracted by one reviewer (RCL), with cross-verification by another (KL, KM). Discrepancies were resolved through discussion. We extracted data on study design, population, location, employment sector, working conditions, and health outcomes. To avoid duplication, when multiple publications reported on the same outcomes in the same population, only the most comprehensive study was included in the meta-analysis.

Studies were categorised based on their health focus through a narrative synthesis of health outcomes, accompanied by a descriptive analysis of these outcomes and associated factors. Comparisons between migrant and local workers, and between transnational and non-migrant families, were made where data permitted. Health risks among migrant workers and their transnational families were integrated into a multi-layered conceptual framework.

We used a random-effects model in R (version 4.4.2) to calculate pooled morbidity prevalence and 95% confidence intervals among migrant workers and transnational families. Forest plots were generated for both prevalence and relative risk estimates, the latter based on studies with comparator groups (local workers or non-migrant households). Heterogeneity was assessed using the *I*^*2*^ statistic. The use of a random-effects model was guided by narrative synthesis indicating substantial heterogeneity across study characteristics. Sensitivity analyses excluded studies rated as low or average quality to assess robustness of pooled estimates.

The quality of included studies was assessed using the Joanna Briggs Institute Checklist for Prevalence Studies,^20^ which evaluates risk of bias, methodological rigour, and reporting transparency. Studies scoring less than 50% were classified as low quality, 50–70% as average quality, and above 70% as high quality. For mixed-methods studies, quality was assessed using an integrated approach combining criteria for cross-sectional and qualitative studies. No studies were excluded based on quality; however, quality ratings informed the interpretation of study findings. All studies were independently assessed by one reviewer (RCL) and cross-checked by another (KL, KM, MS, MB, BO, CK, AI), with discrepancies resolved by consensus.

### Role of funding source

The funder of the study had no role in study design, data collection, data analysis, data interpretation, writing of the report, or the decision to submit for publication. All authors had full access to all study data and were responsible for the decision to submit for publication.

## Results

### Characteristics of included studies

**Fig. 1:**
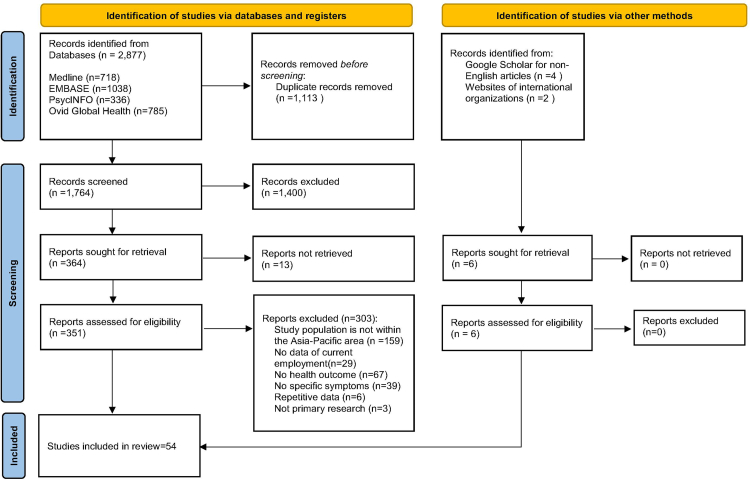
Study selection.

A total of 2877 publications were identified, of which 1113 were duplicates. After removal, 1764 articles were screened by title and abstract. Of these, 351 were selected for full-text review, and 54 studies were included in the final analysis (Fig. 1). These studies collectively included data from 86,620 individuals, comprising 64,172 migrant workers and 22,448 transnational family members. The characteristics of the included studies are summarised in Table 1. Of these, 48 were peer-reviewed articles published in English,^21^, ^22^, ^23^, ^24^^,^^26^, ^27^, ^28^, ^29^, ^30^, ^31^, ^32^, ^33^, ^34^, ^35^^,^^37^, ^38^, ^39^, ^40^, ^41^, ^42^, ^43^^,^^45^, ^46^, ^47^, ^48^^,^^50^, ^51^, ^52^, ^53^, ^54^, ^55^, ^56^, ^57^, ^58^, ^59^, ^60^^,^^62^, ^63^, ^64^, ^65^, ^66^, ^67^, ^68^^,^^70^, ^71^, ^72^, ^73^, ^74^ while four were published in Japanese,^25^ Korean,^36^ traditional Chinese,^49^ and simplified Chinese.^61^ Two reports—one from ILO^44^ and one from the IOM^69^—were also included.

**Table 1:** Characteristics of included studies on migrant workers and transnational families in the Asia–Pacific region.

Study	Year	Country of origin (migrant worker)	Sex ratio (%) (male/female)	Employment sector	Health outcome	Study design	Quality score^a^ (%)
Aryal et al.^21^	2019	Nepal	0/100	Not applicable	Gynaecological diseases, depression	Cross-sectional	63
Aryal et al.^22^	2020	Nepal	0/100	Not applicable	Depression	Cross-sectional	75
Amit et al.^23^	2020	Philippines	70/30	Manufacturing	Musculoskeletal disorders	Cross-sectional	63
Asri and Chuang^24^	2023	Indonesia	47/53	Unknown	Depression	Cross-sectional	88
Aida et al.^25^	2023	Indonesia, Vietnam, Philippines, etc.	56/44	Nursing, manufacturing, processing, construction	Mixed health outcomes	Case series	90
Cheung et al.^26^	2019	Philippines	0/100	Domestic worker	Depression	Cross-sectional	75
Chen and Luo^27^	2023	Thailand	93/7	Manufacturing	Musculoskeletal disorders	Cross-sectional	88
Dutta^28^	2017	Bangladesh	Not available	Construction	Occupational injuries and illnesses	Qualitative study	80
Gao et al.^29^	2014	Indonesia	0/100	Domestic	Dental caries	Cross-sectional	100
Graham et al.^30^	2015	Indonesia, Vietnam, Philippines	32/68	Not applicable	Common Mental Disorders	Mixed methods	72
Hall et al.^31^	2019	Philippines	0/100	Domestic worker	Depression and anxiety	Cross-sectional	88
Hall et al.^32^	2019	Philippines	0/100	Domestic worker	Mixed health outcomes	Qualitative study	70
Hnuploy et al.^33^	2019	Myanmar	58/42	Manufacturing, agriculture, fishing, construction, service, domestic worker	Depression	Cross-sectional	88
Habib et al.^34^	2020	Syria	52/48	Agriculture, construction, manufacturing	Musculoskeletal disorders	Cross-sectional	100
Habib et al.^35^	2021	Syria	52/48	Agriculture, construction, manufacturing	Occupational injuries and illnesses	Cross-sectional	100
Jo et al.^36^	2009	China, Sri Lanka, Bangladesh, Myanmar, etc.	78/22	Manufacturing, construction, service, care worker	Musculoskeletal disorders	Cross-sectional	100
Jayatissa and Wickramage^37^	2016	Sri Lanka	53/47	Not applicable	Stunting, wasting and underweight	Cross-sectional	88
Knipe et al.^38^	2019	Sri Lanka	Not available	Not applicable	Suicide attempt	Cohort study	64
Kesornsri et al.^39^	2019	Myanmar	56/44	Manufacturing, fishing and processing	Depression and anxiety	Cross-sectional	88
Kang et al.^40^	2019	China, Indonesia, Vietnam, Philippines	87/13	Fishing	Mixed health outcomes	Case series	60
Kunwar et al.^41^	2020	Nepal	53/47	Not applicable	Stunting, wasting and underweight	Cross-sectional	100
Kim et al.^42^	2022	Vietnam, Cambodia	44/56	General worker, service, agriculture	Depression	Cross-sectional	100
Kwon et al.^43^	2023	Vietnam	100/0	Agriculture, construction, fishing, manufacturing	Mixed health outcomes	Cross-sectional	50
Lee et al.^44^	2011	China, Vietnam, Sri Lanka, Indonesia, etc.	30/70	Construction, manufacturing	Occupational injuries	Narrative text	100
Lee et al.^45^	2015	China	64/36	Domestic, service, manufacturing, construction	Occupational injuries and illnesses	Cross-sectional	63
Lee et al.^46^	2016	China	0/100	Not available	Cardiovascular diseases	Cross-sectional	65
Labao et al.^47^	2018	Philippines	10/90	Domestic worker, service, manufacturing	Musculoskeletal disorders	Cross-sectional	75
Lee and Cho^48^	2019	China	81/19	Manufacturing, construction, agriculture, service	Occupational injuries and death	Case series	90
Liu et al.^49^	2023	Taiwan	69/31	Agriculture, manufacturing, construction, service	Mixed health outcomes	Cross-sectional	100
Meyer et al.^50^	2016	Myanmar	33/66	Agriculture, manufacturing, sex worker	Depression, anxiety	Cross-sectional	100
Mohammed and Kosalram^51^	2022	India	100/0	Construction, skilled worker, service	Diabetes, Cardiovascular diseases	Cross-sectional	50
Nakagawa et al.^52^	2021	Cambodia	22/78	Food processing	Parasite infection	Case report	100
Palupi et al.^53^	2017	Indonesia	0/100	Domestic worker	Fatigue, depression	Cross-sectional	100
Pradhan et al.^54^	2019	Nepal	Not available	Construction	Cardiovascular diseases	Case series	30
Paudya et al.^55^	2020	Nepal	Not available	Not available	Mixed health outcomes	Systematic review	64
Santos et al.^56^	2015	Sri Lanka, Indonesia, India, Napal, etc.	48/52	Manufacturing	Musculoskeletal disorders	Cross-sectional	63
Siriwardhana et al.^57^	2015	Sri Lanka	27/73	Not applicable	Common Mental Disorders	Cross-sectional	100
Soe et al.^58^	2015	Myanmar	32/68	Manufacturing	Musculoskeletal disorders	Cross-sectional	100
Sato et al.^59^	2019	Indonesia	20/80	Nursing	Mental health problem	Cross-sectional	75
Shah et al.^60^	2020	India, Pakistan, Bangladesh	100/0	Manual labour, driver, office work	Diabetes	Cross-sectional	88
Su et al.^61^	2021	China	96/4	Construction, office work	Musculoskeletal disorders	Cross-sectional	88
Spitzer et al.^62^	2023	Indonesia, Philippines	0/100	Domestic worker	Mixed health outcomes	Qualitative	90
Sucipto et al.^63^	2023	Indonesia	Not available	Not applicable	Stunting, malnutrition, mental health problems	Cross-sectional	40
Sumerlin et al.^64^	2024	Indonesia, Philippines	0/100	Domestic worker	Depression and anxiety	Cross-sectional	100
Thetkathuek and Jaide^65^	2017	Cambodia	58/42	Agriculture	Occupational injuries and illnesses	Cross-sectional	63
Thetkathuek et al.^66^	2018	Cambodia	58/42	Agriculture	Musculoskeletal disorders	Cross-sectional	88
Thetkathuek et al.^67^	2020	Cambodia	61/39	Agriculture	Occupational injuries and illnesses	Cross-sectional	100
Wickramage et al.^68^	2015	Sri-Lanka	40/60	Not applicable	Underweight, mental health problems	Cross-sectional	88
Wickramage et al.^69^	2015	Bangladesh, India, Indonesia, Nepal, etc.	Not available	Not applicable	Underweight, psychiatric diagnosis, Common Mental Disorders	Narrative text	100
Wongsanuphat et al.^70^	2019	Myanmar	Not available	Manufacturing	Measles	Cross-sectional	63
Winata and McLafferty^71^	2023	Indonesia	0/100	Domestic worker	Mixed health outcomes	Mixed methods	78
Yi et al.^72^	2019	Philippines	0/100	Domestic worker	Gambling disorder	Cross-sectional	100
Yi et al.^73^	2021	Bangladesh, India, China, Myanmar	Not available	Construction, manufacturing, skilled worker	Occupation-related illnesses, body pains, cold-like symptoms	Cross-sectional	50
Zerguine et al.^74^	2018	Indonesia, Bangladesh, Pakistan, etc.	100/0	Construction	Work-related injuries	Cross-sectional	63

a Study quality was evaluated utilising a 100 percent scale: <50% were defined low quality, 50–70% were average, and <70 as high quality.

Of the 54 included studies, 39 (72%)^21^, ^22^, ^23^, ^24^^,^^26^^,^^27^^,^^29^, ^30^, ^31^^,^^33^, ^34^, ^35^^,^^37^^,^^39^^,^^41^^,^^43^^,^^45^^,^^47^^,^^49^^,^^51^^,^^53^^,^^56^, ^57^, ^58^, ^59^^,^^61^^,^^64^, ^65^, ^66^, ^67^^,^^70^^,^^72^, ^73^, ^74^ were cross-sectional. Most were high quality, with a median quality score of 88%, with 37 (69%) rated as high, and only two rated low. One case series received a score of 30%, due to unclear reporting of the participants' health conditions.^54^ Another qualitative study scored 40%, largely because of unclear linkages between the research methodology, study population, and analytical approach.^63^ Detailed quality assessment scores are presented in Table 1.

Of the 54 included studies, 44 articles^23^, ^24^, ^25^, ^26^, ^27^, ^28^, ^29^^,^^31^, ^32^, ^33^, ^34^, ^35^, ^36^^,^^39^^,^^40^^,^^42^, ^43^, ^44^, ^45^, ^46^, ^47^, ^48^, ^49^, ^50^, ^51^, ^52^, ^53^, ^54^, ^55^, ^56^^,^^58^, ^59^, ^60^, ^61^, ^62^^,^^64^, ^65^, ^66^, ^67^^,^^70^, ^71^, ^72^, ^73^, ^74^ focused on labour migration, while 10 articles^21^^,^^22^^,^^30^^,^^37^^,^^38^^,^^41^^,^^57^^,^^63^^,^^68^^,^^69^ examined transnational families. Data were collected across 17 countries and territories. Among the 54 included studies, 51 reported study locations, with nearly equal distribution between high-income countries (24 studies; 47%) and middle-income countries (27 studies; 53%). The study locations included: Thailand (n = 8),^33^^,^^39^^,^^50^^,^^58^^,^^65^, ^66^, ^67^^,^^70^ Korea (n = 7),^23^^,^^36^^,^^42^^,^^43^^,^^45^^,^^46^^,^^48^ Sri Lanka (n = 5),^37^^,^^38^^,^^57^^,^^61^^,^^68^ Hong Kong (n = 4),^26^^,^^30^^,^^64^^,^^65^ Malaysia (n = 4),^47^^,^^56^^,^^62^^,^^74^ Taiwan (n = 4),^24^^,^^27^^,^^40^^,^^53^ Nepal (n = 4),^21^^,^^22^^,^^41^^,^^55^ Japan (n = 3),^25^^,^^52^^,^^59^ Macao (n = 3),^31^^,^^32^^,^^72^ Lebanon (n = 2),^34^^,^^35^ India (n = 1),^51^ Indonesia (n = 1),^63^ Qatar (n = 1),^54^ Singapore (n = 1),^73^ United Arab Emirates (n = 1),^60^ and Vietnam (n = 1).^49^ One additional study^30^ reported multiple locations across Indonesia, the Philippines, and Vietnam. The classification of income groups was based on World Bank standards.^75^

The included studies indicated that migration primarily flowed from middle-income to high-income countries. Among the 18 labour-sending countries, 16 were classified as middle-income, while 12 of the 17 labour-receiving countries were high-income. Notably, five countries—Taiwan, Thailand, Malaysia, Vietnam, and Sri Lanka—functioned as both labour-sending and -receiving countries, reflecting the dynamic and bidirectional nature of labour mobility in the region. Details of these classifications are summarised in Table 2.

**Table 2:** Labour migration flow in the Asia–Pacific region, classified by country role and World Bank income group.

**Labour-sending countries (n = 18)**	
High income (n = 1)	Taiwan
Middle income (n = 16)	Bangladesh, Cambodia, China, India, Indonesia, Kazakhstan, Malaysia, Mongolia, Myanmar, Nepal, Pakistan, Philippines, Sri Lanka, Thailand, Uzbekistan, Vietnam
Low income (n = 1)	Syria
**Labour-receiving countries (n** = **17)**	
High income (n = 12)	Oman, Bahrain, Hong Kong, Japan, Korea, Kwait, Macao, Saudi Arabia, Singapore, Taiwan, United Arab Emirates, Qatar
Middle income (n = 5)	Lebanon, Malaysia, Sri Lanka, Thailand, Vietnam
**Labour-sending and receiving countries (n** = **5)**	
High income (n = 1)	Taiwan
Middle income (n = 4)	Thailand, Malaysia, Sri Lanka, Vietnam

Countries included in the search strategy but not listed here (e.g. Tonga, Yemen) had no eligible studies identified.

Migrant workers in this region were predominantly male (43,117 [71%]). More than 75% were employed in manufacturing (19,056 [35%]), construction (15,424 [28%]), and service sectors (7629 [14%]) including drivers, restaurant workers, car washers and sales assistants. Other sectors included agriculture (6762 [12%]) and domestic work (3834 [7%]).

### Health issues of migrant workers

Among the 44 studies on migrant workers, seven key health focus areas were identified: mental health (n = 11),^24^^,^^26^^,^^31^^,^^33^^,^^39^^,^^42^^,^^50^^,^^53^^,^^59^^,^^64^^,^^72^ musculoskeletal disorders (MSDs) (n = 9),^23^^,^^27^^,^^34^^,^^36^^,^^47^^,^^56^^,^^58^^,^^61^^,^^66^ mixed health outcomes (referring to studies that concurrently examined a range of physical and psychological health issues) (n = 8),^25^^,^^32^^,^^40^^,^^43^^,^^49^^,^^55^^,^^62^^,^^71^ occupational injuries and illnesses (n = 8),^28^^,^^35^^,^^44^^,^^45^^,^^48^^,^^65^^,^^67^^,^^74^ diabetes and cardiovascular diseases (CVDs) (n = 4),^46^^,^^51^^,^^54^^,^^60^ infectious diseases (n = 3),^52^^,^^70^^,^^73^ and oral health (n = 1).^29^

Occupational injuries and illnesses exhibited the highest prevalence, affecting 45,661 migrant workers (88%) who reported at least one work-related morbidity in our review. Of note, 85% of these cases were reported in a single study of Chinese migrant workers in South Korea.^48^ When this study was excluded, occupational injuries and illnesses remained the most frequently reported health condition, affecting 3572 individuals. The most common type of occupational injury was cuts and wounds (n = 955). Other major reported health conditions included mental health issues (1975 [3.8%]), MSDs (1973 [3.8%]), and diabetes and CVDs (745 [1.4%]). Two studies reported migrant worker mortality, including 353 deaths caused by collisions^48^ and 571 deaths attributed to CVDs.^54^

**Fig. 2:**
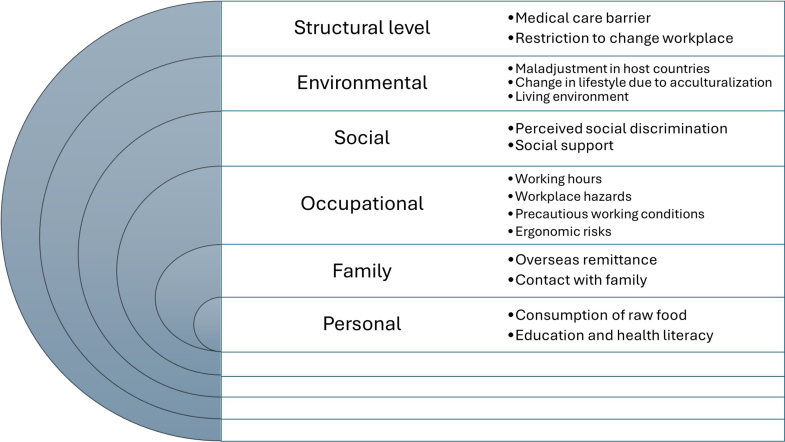
Health risk factors of migrant workers and transnational families in the Asia–Pacific region. References: Medical care barrier^26^^,^^32^^,^^36^^,^^44^^,^^70^; Restriction to change workplace^44^^,^^48^; Maladjustment in host countries^25^^,^^38^^,^^46^^,^^49^; Change in lifestyle due to acculturation^60^; Living environment^28^^,^^30^^,^^70^^,^^73^; Perceived social discrimination^31^; Social support^26^^,^^42^^,^^44^^,^^56^^,^^72^; Working hours^24^^,^^26^^,^^28^^,^^39^^,^^41^^,^^54^^,^^55^^,^^62^, ^63^, ^64^^,^^66^, ^67^, ^68^^,^^71^; Workplace hazards^35^^,^^43^^,^^45^^,^^48^^,^^51^^,^^54^^,^^55^^,^^66^^,^^74^; Working conditions^26^^,^^28^^,^^48^^,^^50^^,^^52^^,^^62^^,^^70^; Ergonomic risks^25^^,^^33^^,^^43^^,^^50^^,^^59^^,^^60^^,^^73^; Overseas remittance^22^^,^^30^^,^^41^^,^^57^^,^^68^; Contact with family^22^^,^^24^^,^^56^; Consumption of raw food^55^; Education and health literacy.^37^^,^^57^^,^^63^

Through thematic analysis of reported health risks, we categorised these into six interrelated levels—personal, familial, occupational, social, environmental, and structural—illustrating the complex, multi-layered determinants of health among migrant workers (Fig. 2). Among these, occupational factors were the most pervasive and consistently reported source of risk. Three key occupational factors—long working hours, workplace hazards, and precarious employment—were most frequently cited as contributing to multiple and overlapping health burdens among migrant workers.

Long working hours, including overwork, lack of breaks, and no rest days—were cited in 14 studies.^24^^,^^28^^,^^33^^,^^49^^,^^51^^,^^54^^,^^56^^,^^58^^,^^62^^,^^64^, ^65^, ^66^, ^67^^,^^71^ These conditions were associated with increased risks of mental health issues,^24^^,^^33^^,^^64^ MSDs,^56^^,^^58^^,^^66^ diabetes and CVDs,^51^^,^^54^ mixed health outcomes,^49^^,^^62^^,^^71^ and occupational injuries and illnesses.^28^^,^^65^^,^^67^ Workplace hazards including exposure to heat, chemicals, dust, sharp or heavy objects, collisions, and lack of protective equipment were described in 11 studies.^35^^,^^43^, ^44^, ^45^^,^^48^^,^^51^^,^^54^^,^^56^^,^^65^^,^^67^^,^^74^^,^ These hazards contributed to MSDs,^56^ mixed health outcomes,^43^ diabetes and CVDs,^51^^,^^54^ and occupational injuries and illnesses.^35^^,^^44^^,^^45^^,^^48^^,^^65^^,^^67^^,^^74^ Precarious working conditions, including physical and verbal abuse, job stress, and lack of workplace control, were reported in seven studies.^26^^,^^28^^,^^35^^,^^42^^,^^50^^,^^56^^,^^62^ These factors were associated with increased risks of mental health issues,^26^^,^^42^^,^^50^ MSDs,^56^ mixed health outcomes,^62^ and occupational injuries and illnesses.^28^^,^^33^ Barriers to medical care were identified in five studies.^26^^,^^32^^,^^36^^,^^44^^,^^70^ These barriers were associated with worsen health conditions, including MSDs,^36^ measles infection,^70^ mixed health outcomes,^32^ and occupational injuries and illnesses.^28^^,^^44^

Together, these findings underscore the multifaceted and interlinked nature of occupational determinants, which expose migrant workers not only to direct physical and psychological harm but also to chronic health inequalities driven by excessive labour demands, hazardous environments, and systemic barriers to care.

### Health issues of transnational families

Ten studies on transnational families included left-behind children, stay-behind spouses, older adults, and caregivers with some examining multiple generations. Three main health focus areas were identified: mental health (n = 4),^22^^,^^30^^,^^38^^,^^57^ malnutrition (n = 2),^37^^,^^41^ and mixed health outcomes involving both mental health and nutritional status (n = 4).^21^^,^^63^^,^^68^^,^^69^ In total, 50.1% reported mental health issues—including anxiety, depression, suicide attempts, and other common disorders (n = 1520)—and 30.4% reported malnutrition, including stunting, wasting, and underweight (n = 954).

The health of transnational families was associated with the frequency of remittances from migrant workers^22^^,^^30^^,^^41^^,^^57^^,^^68^ and their own education levels.^37^^,^^57^^,^^63^ Given the interconnected health fluctuations between transnational families and migrant workers, these factors could be incorporated into the framework of multiple risk factors affecting migrant workers (Fig. 2).

### Meta-analysis: work-related morbidity of migrant workers

**Fig. 3:**
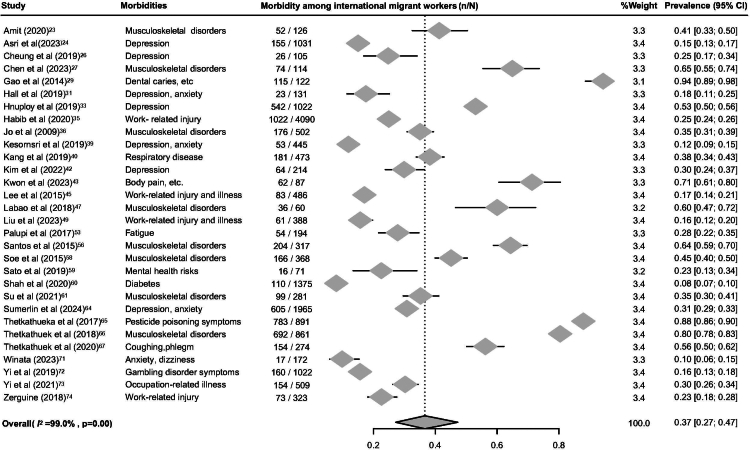
Forest plot of prevalence of having at least one reported work-related morbidity among migrant workers in the Asia–Pacific region.

**Fig. 4:**
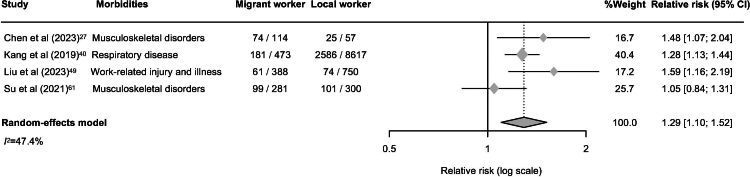
Forest plot of relative risk of having at least one reported work-related morbidity among migrant workers compared with local workers.

The meta-analysis of work-related morbidity among migrant workers included data from 18,019 individuals across 30 studies.^23^^,^^24^^,^^26^^,^^27^^,^^29^^,^^31^^,^^33^^,^^35^^,^^36^^,^^39^^,^^40^^,^^42^^,^^43^^,^^45^^,^^47^^,^^49^^,^^53^^,^^56^^,^^58^, ^59^, ^60^, ^61^^,^^64^, ^65^, ^66^, ^67^^,^^71^, ^72^, ^73^, ^74^ The pooled prevalence of reporting at least one work-related morbidity was 37% (95% CI 27–47; *I*^*2*^ = 99.0%; Fig. 3), indicating that more than one in three migrant workers experience work-related health problems. Of the 30 studies, four were included in the meta-analysis of relative risk, selected based on methodological homogeneity in employment sectors and health outcomes. Based on four studies^27^^,^^40^^,^^49^^,^^61^ involving 1256 migrant workers and 9724 local workers, the pooled relative risk of work-related morbidity was 1.29 (95% CI: 1.10–1.52; *I*^*2*^ = 47.4%; Fig. 4), suggesting that migrant workers are about 30% more likely to report morbidity compared with native workers.

### Meta analysis: morbidity of transnational families

**Fig. 5:**
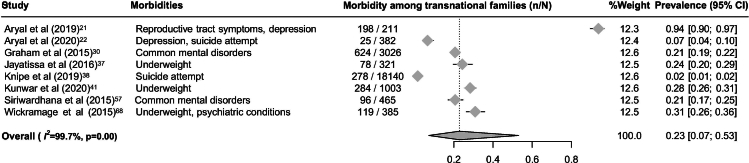
Forest plot of prevalence of having at least one reported morbidity associated with labour migration among transnational families in the Asia–Pacific region.

**Fig. 6:**
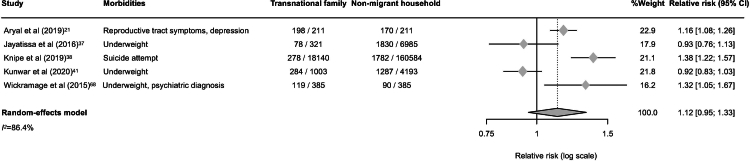
Forest plot of relative risk of having at least one reported morbidity associated with labour migration among transnational families compared with non-migrant households.

A total of 23,933 transnational family members from eight studies^21^^,^^22^^,^^30^^,^^37^^,^^38^^,^^41^^,^^57^^,^^68^ were included in the meta-analysis. The pooled prevalence of reporting at least one morbidity, including mental health conditions and malnutrition, associated with labour migration was 23% (95% CI: 7–53; *I*^*2*^ = 99.7%; Fig. 5), highlighting a substantial health burden among left-behind family members. To compare health outcomes between transnational families and non-migrant households, a separate meta-analysis was conducted using five studies,^21^^,^^37^^,^^38^^,^^41^^,^^68^ selected based on comparable characteristics in country of origin and health outcome focus. The pooled relative risk was 1.12 (95% CI: 0.95–1.33; *I*^*2*^ = 86.4%; Fig. 6), indicating only a modest and statistically non-significant elevation in morbidity among transnational families.

Sensitivity analyses assessed the impact of study quality on pooled estimates. Among migrant workers, excluding low and average quality studies yielded a prevalence of 33% (95% CI: 0.23–0.45; *I*^*2*^ = 98.9%) and a relative risk of 1.32 (95% CI: 1.01–1.74; *I*^*2*^ = 64.9%), consistent with main findings (Appendix pp 4–5). For transnational families, the pooled prevalence was 21% (95% CI: 0.13–0.30; *I*^*2*^ = 94.4%), and the relative risk compared with non-migrant households was 1.03 (95% CI: 0.83–1.27; *I*^*2*^ = 74.1%), showing no notable deviation (Appendix pp 6–7).

## Discussion

This large multi-country dataset identified substantial occupational health risks among international migrant workers in the Asia–Pacific region. Long working hours, hazardous conditions, precarious employment, and limited healthcare access were consistently associated with musculoskeletal disorders, occupational injuries, depression, gambling disorders, and cardiovascular disease. Although some studies support the “healthy migrant effect,”^4^^,^^27^ this advantage diminishes over time due to cumulative exposure to occupational and structural stressors.^26^^,^^49^^,^^54^^,^^61^^,^^70^^,^^72^

Our findings align with global reviews reporting high occupational morbidity among migrant workers,^2^, ^3^, ^4^ though our pooled prevalence (37%) was slightly lower—likely due to underreporting and limited disclosure.^4^^,^^5^ The relative risk (1.29) was consistent with global estimates,^3^ but the absence of regional mortality data limited comparisons for fatal injuries. For example, during a measles outbreak in Korea, migrant workers had twice the attack rate of local workers (1.4% vs 0.7%), attributed to lower vaccination coverage (11.6% vs 36.7%).^70^

For transnational families, findings indicate elevated risks of mental health issues and undernutrition, in line with global reviews of left-behind children.^5^^,^^6^ However, the pooled relative risk (1.12) was only marginally elevated, possibly reflecting differences in population composition and limited disaggregated data. Many studies included not only children, but also spouses and older adults. Emotional strain and irregular remittances were key contributors to poor health outcomes.^22^^,^^30^^,^^41^^,^^57^^,^^62^^,^^68^ Some studies that combined internal and international migrant households were excluded from our analysis due to the lack of disaggregated data. For example, a cross-sectional study from Cambodia reported higher morbidity among both adult members and left-behind children in migrant families compared to non-migrant households.^76^ Although not eligible for inclusion in the meta-analysis, such evidence underscores the potential health vulnerabilities of transnational family members and may partly explain why the pooled relative risk in our review was only modestly elevated.

This review provides the first region-specific morbidity estimates based on multilingual evidence. However, most included studies were cross-sectional and relied on self-reported data. Female migrant workers beyond the domestic sector were also underrepresented. This underrepresentation is evident when compared with ILO estimates, which show that male and female migrant workers contribute nearly equally to the labour force in the Asia–Pacific region,^1^ whereas men accounted for 71% of participants in our included studies. Publication bias cannot be excluded, and substantial heterogeneity reflects differences in study populations and outcome measures. While this limits the precision of pooled estimates, forest plots were retained to illustrate the overall distribution and direction of effects across the region. Beyond these methodological considerations, it is also important to note that while occupational factors were the most consistently reported and therefore emphasised as key contributors in the main text, our thematic analysis (Fig. 2) also highlighted other contextual risks such as living conditions. For example, limited facilities in migrant worker housing have been linked to COVID-19 exposure risks in Singapore.^73^

These health burdens stem not from migration itself, but from inadequate protections—affecting both workers and their families across borders. Strengthening protections in both sending and receiving countries is therefore essential. Although 80% of Asia–Pacific countries have bilateral labour migration agreements, only 44% have implemented ethical recruitment measures, and fewer than half have mechanisms to safeguard the rights of their overseas workers.^77^ In the 2023 report on migration and health, the Association of Southeast Asian Nations (ASEAN) Secretariat called on member states to ensure health coverage for migrant workers by 2025, while at the same time acknowledging that workers continue to face substantial barriers to accessing care, including language difficulties, financial constraints, frequent changes in employment, and exclusion from local insurance systems.^78^ These contrasting messages underscore the urgent need for strengthened cooperation between labour-sending and labour-receiving countries.

Regional mechanisms already exist to facilitate such cooperation. For example, bilateral agreements between labour-sending and receiving countries in the Asia–Pacific have included explicit health protections for migrant workers. The Bangladesh–Qatar Agreement (2008) stipulates that employers must cover medical treatment free of charge for workers, while the Nepal–Jordan General Agreement (2017) requires employers to provide all necessary medical care.^79^ In addition, two active consultative processes in the region involve governments at different ends of the migration corridor: the Colombo Process, a forum of labour-sending countries in South and South-East Asia,^80^ and the Abu Dhabi Dialogue, which brings together Colombo Process countries of origin with Gulf Cooperation Council destination countries.^81^ These mechanisms provide platforms for dialogue and cooperation to promote the welfare and well-being of migrant workers. Our findings may contribute to evidence-based policy development within these regional frameworks to reduce the health risks associated with labour migration.

By 2023, the outflow of migrant workers from Asia–Pacific countries had risen by 33% from 2022, while inflows and migrant stocks in major destinations surpassed pre-pandemic levels.^77^ This trend underscores the region's growing dependence on migrant labour and the urgent need to protect worker health to sustain economic stability. Despite its central role in global labour migration, the Asia–Pacific remains underrepresented in global policy frameworks. Aligning national policies with WHO's Global Plan of Action, SDG 8.8, and the Global Compact for Safe, Orderly and Regular Migration can support regional action to improve safety, promote ethical recruitment, and expand healthcare access.

Ultimately, this review provides the first region-wide synthesis of migrant worker health in the Asia–Pacific, addressing a major evidence gap and highlighting substantial but preventable adverse health outcomes. Strengthening protections and closing policy gaps through coordinated regional action, involving governments, health services, and employers, is essential to safeguard the health of both migrant workers and their families, with direct implications for sustainable economic development.

## Contributors

SH and RCL conceived the study. RCL developed the protocol with input from KL, KM, UT, AK, CZ, and SH. RCYL conducted the database search, abstract screening, data extraction, and quality appraisal, which were cross-verified by KL, KM, NRM, MS, MB, BO, CK, and AI. RCL performed the data analysis and drafted the manuscript, with contributions from all authors.

## Data sharing statement

No additional data are available.

## Declaration of interests

We declare no competing interests.

## Figures and Tables

**Appendix 4:**
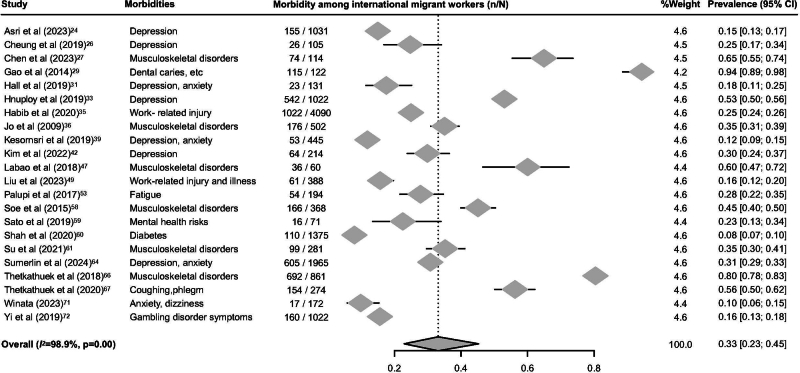
Sensitivity analysis of prevalence of at least reported one work-related morbidity of migrant workers excluding studies of middle and low quality.

**Appendix 5:**
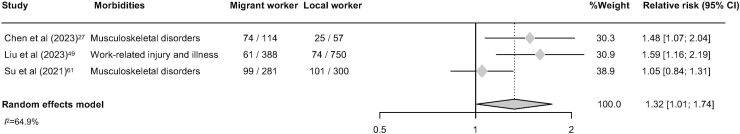
Sensitivity analysis of relative risk of having at least one reported work-related morbidity associated in migrant workers compared with local workers excluding studies of middle and low quality.

**Appendix 6:**
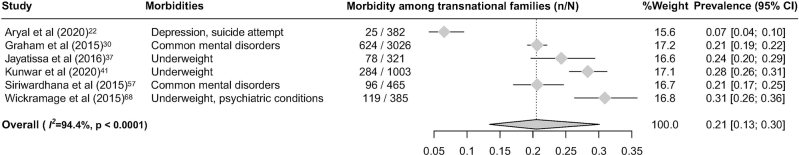
Sensitivity analysis of prevalence of at least reported one morbidity of transnational families associated with labour migration excluding studies of middle and low quality.

**Appendix 6:**
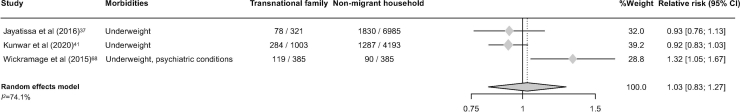
Sensitivity analysis of relative risk of having at least one reported morbidity associated with labour migration in transnational families compared with non-migrant households excluding studies of middle and low quality.
