# Corrigendum to “Adverse health outcomes among migrant workers and transnational families in the Asia–Pacific: a systematic review and meta-analysis” [The Lancet Regional Health–Western Pacific 2025;64: 101720]

**DOI:** 10.1016/j.lanwpc.2025.101774

**Published:** 2025-12-17

**Authors:** Rosita Chia-Yin Lin, Karen Lau, Kathryn Mackey, Natasha Roya Matthews, Maushmi Selvamani, Beatriz Morais, Oumnia Bouaddi, Chaelin Kim, Azusa Iwamoto, Masami Fujita, Ursula Trummer, Tran Ngoc Dang, Alena Kamenshchikova, Cathy Zimmerman, Sally Hargreaves

**Affiliations:** a The Migrant Health Research Group and the Consortium for Migrant Worker Health, The School of Health and Medical Sciences, Institute for Infection and Immunity, City St George's, University of London, London, UK; b Department of Global Health & Development, London School of Hygiene & Tropical Medicine, London, UK; c Department of Infectious Disease Epidemiology and Dynamic, London School of Hygiene & Tropical Medicine, London, UK; d Emergency Department Clinical Research Group, St George's University Hospitals NHS Foundation Trust, London, UK; e Schizophrenia Research Foundation India (SCARF Hospital), Chennai, India; f Mohammed VI International School of Public Health, Mohammed VI University of Sciences and Health, Casablanca & Mohammed VI Center for Research and Innovation (CM6RI), Rabat, Morocco; g Bureau of Global Health Cooperation, Japan Institute for Health Security, Tokyo, Japan; h Centre for Health and Migration, Vienna, Austria; i Faculty of Public Health, University of Medicine and Pharmacy at Ho Chi Minh City, Viet Nam; j Department of Health, Ethics and Society, Maastricht University, Maastricht, Netherlands; k Department of Social Medicine, Maastricht University, Maastricht, Netherlands; l Department of Medical Microbiology, Infectious Diseases and Infection Prevention, Maastricht University, Maastricht, Netherlands

The authors regret to inform that the first author's name and surname have been reversed for the below 2 authors which is corrected in this correction.

Morais Beatriz should be corrected as *Beatriz Morais*.

Bouaddi Oumnia should be corrected to *Oumnia Bouaddi*.

There is a Typographical error in Figure 2.

In the “occupational” category, the term “precautious working conditions” is incorrect.

It should be corrected to “precarious working conditions”.

**Figure:**
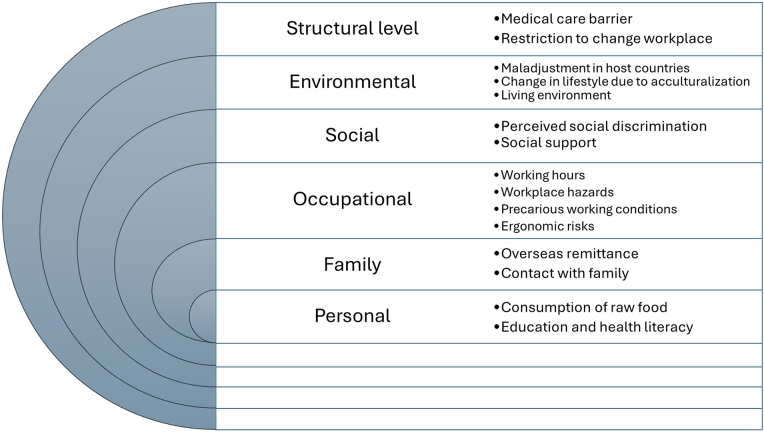

There is a minor correction in the Footnote of Table 1.

The current text reads:

“<50% were defined low quality, 50–70% were average, and <70 as high quality.”

The corrected version should be:

“<50% were defined as low quality, 50–70% as average, and >70 as high quality.”

The authors would like to apologise for any inconvenience caused.

