# Supplementary material for: Adverse health outcomes among migrant workers and transnational families in the Asia–Pacific: a systematic review and meta-analysis
Source: Lancet Reg Health West Pac. 2025 Oct 28;64:101720. doi: 10.1016/j.lanwpc.2025.101720 (PMC12597289; doi:10.1016/j.lanwpc.2025.101720)
Supplement: Appendix 1-3 [file mmc1.docx]

**Table 1** Characteristics of included studies

| **Study** | **Year** | **Country of origin**  **(migrant worker)** | **Sex ratio(%)**  **(male/female)** | **Employment sector** | **Health outcome** | **Study design** | **Quality score(%)** |
| --- | --- | --- | --- | --- | --- | --- | --- |
| Aryal et al^21^ | 2019 | Nepal | 0/100 | Not applicable | Gynaecological diseases, depression | Cross-sectional | 63 |
| Aryal et al^22^ | 2020 | Nepal | 0/100 | Not applicable | Depression | Cross-sectional | 75 |
| Amit et al^23^ | 2020 | Philippines | 70/30 | Manufacturing | Musculoskeletal disorders | Cross-sectional | 63 |
| Asri and Chuang^24^ | 2023 | Indonesia | 47/53 | Unknown | Depression | Cross-sectional | 88 |
| Aida et al^25^ | 2023 | Indonesia, Vietnam, Philippines, etc. | 56/44 | Nursing, manufacturing, processing, construction | Mixed health outcomes | Case series | 90 |
| Cheung et al^26^ | 2019 | Philippines | 0/100 | Domestic worker | Depression | Cross-sectional | 75 |
| Chen and Luo^27^ | 2023 | Thailand | 93/7 | Manufacturing | Musculoskeletal disorders | Cross-sectional | 88 |
| Dutta^28^ | 2017 | Bangladesh | Not available | Construction | Occupational injuries and illnesses | Qualitative study | 80 |
| Gao et al^29^ | 2014 | Indonesia | 0/100 | Domestic | Dental caries | Cross-sectional | 100 |
| Graham et al^30^ | 2015 | Indonesia, Vietnam, Philippines | 32/68 | Not applicable | Common Mental Disorders | Mixed methods | 72 |
| Hall et al^31^ | 2019 | Philippines | 0/100 | Domestic worker | Depression and anxiety | Cross-sectional | 88 |
| Hall et al^32^ | 2019 | Philippines | 0/100 | Domestic worker | Mixed health outcomes | Qualitative study | 70 |
| Hnuploy et al^33^ | 2019 | Myanmar | 58/42 | Manufacturing, agriculture, fishing, construction, service, domestic worker | Depression | Cross-sectional | 88 |
| Habib et al^34^ | 2020 | Syria | 52/48 | Agriculture, construction, manufacturing | Musculoskeletal disorders | Cross-sectional | 100 |
| Habib et al^35^ | 2021 | Syria | 52/48 | Agriculture, construction, manufacturing | Occupational injuries and illnesses | Cross-sectional | 100 |
| Jo et al^36^ | 2009 | China, Sri Lanka, Bangladesh, Myanmar, etc. | 78/22 | Manufacturing, construction, service, care worker | Musculoskeletal disorders | Cross-sectional | 100 |
| Jayatissa and Wickramage^37^ | 2016 | Sri Lanka | 53/47 | Not applicable | Stunting, wasting and underweight | Cross-sectional | 88 |
| Knipe et al^38^ | 2019 | Sri Lanka | Not available | Not applicable | Suicide attempt | Cohort study | 64 |
| Kesornsri et al^39^ | 2019 | Myanmar | 56/44 | Manufacturing, fishing and processing | Depression and anxiety | Cross-sectional | 88 |
| Kang et al^40^ | 2019 | China, Indonesia, Vietnam, Philippines | 87/13 | Fishing | Mixed health outcomes | Case series | 60 |
| Kunwar et al^41^ | 2020 | Nepal | 53/47 | Not applicable | Stunting, wasting and underweight | Cross-sectional | 100 |
| Kim et al^42^ | 2022 | Vietnam, Cambodia | 44/56 | General worker, service, agriculture | Depression | Cross-sectional | 100 |
| Kwon et al^43^ | 2023 | Vietnam | 100/0 | Agriculture, construction, fishing, manufacturing | Mixed health outcomes | Cross-sectional | 50 |
| Lee et al^44^ | 2011 | China, Vietnam,  Sri Lanka, Indonesia, etc. | 30/70 | Construction, manufacturing | Occupational injuries | Narrative text | 100 |
| Lee et al^45^ | 2015 | China | 64/36 | Domestic, service, manufacturing, construction | Occupational injuries and illnesses | Cross-sectional | 63 |
| Lee et al^46^ | 2016 | China | 0/100 | Not available | Cardiovascular diseases | Cross-sectional | 65 |
| Labao et al^47^ | 2018 | Philippines | 10/90 | Domestic worker, service,manufacturing | Musculoskeletal disorders | Cross-sectional | 75 |
| Lee and Cho^48^ | 2019 | China | 81/19 | Manufacturing, construction, agriculture, service | Occupational injuries and death | Case series | 90 |
| Liu et al^49^ | 2023 | Taiwan | 69/31 | Agriculture, manufacturing, construction, service | Mixed health outcomes | Cross-sectional | 100 |
| Meyer et al^50^ | 2016 | Myanmar | 33/66 | Agriculture, manufacturing, sex worker | Depression, anxiety | Cross-sectional | 100 |
| Mohammed and Kosalram^51^ | 2022 | India | 100/0 | Construction, skilled worker, service | Diabetes, Cardiovascular diseases | Cross-sectional | 50 |
| Nakagawa et al^52^ | 2021 | Cambodia | 22/78 | Food processing | Parasite infection | Case report | 100 |
| Palupi et al^53^ | 2017 | Indonesia | 0/100 | Domestic worker | Fatigue, depression | Cross-sectional | 100 |
| Pradhan et al^54^ | 2019 | Nepal | Not available | Construction | Cardiovascular diseases | Case series | 30 |
| Paudya et al^55^ | 2020 | Nepal | Not available | Not available | Mixed health outcomes | Systematic review | 64 |
| Santos et al^56^ | 2015 | Sri Lanka,  Indonesia, India, Napal, etc. | 48/52 | Manufacturing | Musculoskeletal disorders | Cross-sectional | 63 |
| Siriwardhana et al^57^ | 2015 | Sri Lanka | 27/73 | Not applicable | Common Mental Disorders | Cross-sectional | 100 |
| Soe et al^58^ | 2015 | Myanmar | 32/68 | Manufacturing | Musculoskeletal disorders | Cross-sectional | 100 |
| Sato et al^59^ | 2019 | Indonesia | 20/80 | Nursing | Mental health problem | Cross-sectional | 75 |
| Shah et al^60^ | 2020 | India, Pakistan, Bangladesh | 100/0 | Manual labour, driver, office work | Diabetes | Cross-sectional | 88 |
| Su et al^61^ | 2021 | China | 96/4 | Construction, office work | Musculoskeletal disorders | Cross-sectional | 88 |
| Spitzer et al^62^ | 2023 | Indonesia, Philippines | 0/100 | Domestic worker | Mixed health outcomes | Qualitative | 90 |
| Sucipto et al^63^ | 2023 | Indonesia | Not available | Not applicable | Stunting, malnutrition, mental health problems | Cross-sectional | 40 |
| Sumerlin  et al^64^ | 2024 | Indonesia, Philippines | 0/100 | Domestic worker | Depression and anxiety | Cross-sectional | 100 |
| Thetkathuek and Jaide^65^ | 2017 | Cambodia | 58/42 | Agriculture | Occupational injuries and illnesses | Cross-sectional | 63 |
| Thetkathuek et al^66^ | 2018 | Cambodia | 58/42 | Agriculture | Musculoskeletal disorders | Cross-sectional | 88 |
| Thetkathuek et al^67^ | 2020 | Cambodia | 61/39 | Agriculture | Occupational injuries and illnesses | Cross-sectional | 100 |
| Wickramage et al^68^ | 2015 | Sri-Lanka | 40/60 | Not applicable | Underweight, mental health problems | Cross-sectional | 88 |
| Wickramage et al^69^ | 2015 | Bangladesh, India, Indonesia, Nepal, etc. | Not available | Not applicable | Underweight, psychiatric diagnosis, Common Mental Disorders | Narrative text | 100 |
| Wongsanuphat et al^70^ | 2019 | Myanmar | Not available | Manufacturing | Measles | Cross-sectional | 63 |
| Winata and McLafferty^71^ | 2023 | Indonesia | 0/100 | Domestic worker | Mixed health outcomes | Mixed methods | 78 |
| Yi et al^72^ | 2019 | Philippines | 0/100 | Domestic worker | Gambling disorder | Cross-sectional | 100 |
| Yi et al^73^ | 2021 | Bangladesh, India, China, Myanmar | Not available | Construction, manufacturing, skilled worker | Occupation-related illnesses, body pains, cold-like symptoms | Cross-sectional | 50 |
| Zerguine et al^74^ | 2018 | Indonesia, Bangladesh, Pakistan, etc. | 100/0 | Construction | Work-related injuries | Cross-sectional | 63 |

* Study quality was evaluated utilizing a 100 percent scale: <50% were defined low quality, 50-70% were average, and <70 as high quality

**Table 2**Asia-Pacific labour migration flow by country role and income group (World Bank criteria)

| **Labour-sending countries(n=18)** | |
| --- | --- |
| High income (n=1) | Taiwan |
| Middle income(n=16) | Bangladesh, Cambodia China, India, Indonesia Kazakhstan, Malaysia, Mongolia, Myanmar, Nepal, Pakistan, Philippines, Sri Lanka, Thailand, Uzbekistan, Vietnam |
| Low income(n=1) | Syria |
| **Labour-receiving countries(n=17)** | |
| High income (n=12) | Oman, Bahrain, Hong Kong, Japan, Korea, Kwait,  Macao, Saudi Arabia, Singapore, Taiwan,  United Arab Emirates, Quata |
| Middle income(n=5) | Lebanon, Malaysia, Sri Lanka, Thailand, Vietnam |
| **Labour-sending and receiving countries(n=5)** | |
| High income (n=1) | Taiwan |
| Middle income(n=4) | Thailand, Malaysia, Sri Lanka, Vietnam |

*Countries included in the search strategy but not listed here (e.g. Tonga, Yemen) had no eligible studies identified.*

Appendix 1
**Search strategy example for Ovid MEDLINE**

| **Search concept 1: Health outcomes of labour migrants** | |
| --- | --- |
| 1 | (Labo?r migra* OR migrant work* OR (foreign* OR non-native OR transient OR migrant OR refugee OR undocumented OR expatriate)adj2(work*) OR foreign-work* OR immigrant work* OR economic migra* OR economic immigra* OR OR traffick OR international labo?r migra*).mp. |
| 2 | ((Occupation* OR work* OR employ* OR industr* OR minin* OR quarrying OR forestr* OR rubber plantation* OR plantation* OR palm oil OR fisher* OR fishing OR manufacturing OR retail* OR construction OR agricultur* OR farm* OR brick kiln* OR domestic work*)adj3(health OR injur* OR disease* OR hazard* OR exposure* OR accident* OR hygiene OR safety OR medicine OR trauma* OR fatalit* OR death* OR ill* OR syndrom* OR wound* OR fatigue OR risk* OR exposure OR safety OR disabilit* OR morbidit* or mortalit* OR homicid* OR infect* OR disorder* OR pain* OR ache*)).mp. |
| 3 | ((Occupation* OR work* OR employ* OR industr* OR minin* OR quarrying OR forestr* OR rubber plantation* OR plantation* OR palm oil OR fisher* OR fishing OR manufacturing OR retail* OR construction OR agricultur* OR farm* OR brick kiln* OR domestic work*)adj3(respirat* OR musculoskeletal OR cardiovascular OR cancer* OR hypertensi* OR chronic OR obstructive OR hearing loss* OR dermat* OR allerg* OR repetitive injur* OR silicosis OR pneumoconiosis OR puncture* OR laceration OR electrocution* OR fall* OR drowning OR pneumonia OR exhaustion OR broken bone* OR toxic* OR poison* OR puncture OR burn* OR parasite* OR tendon* OR pesticide* OR insecticide OR dehydration OR ocular OR eye* OR asthma OR bronchitis OR pulmonary)).mp. |
| 4 | ((Occupation* OR work* OR employ* OR industr* OR minin* OR quarrying OR forestr* OR rubber plantation* OR plantation* OR palm oil OR fisher* OR fishing OR manufacturing OR retail* OR construction OR agricultur* OR farm* OR brick kiln* OR domestic work*)adj3(mental* OR psychosocial OR neurotic OR mood)adj3(problem* OR disorder* OR ill* OR health OR stress* OR wellbeing OR well-being)).mp. |
| 5 | ((Occupation* OR work* OR employ* OR industr* OR minin* OR quarrying OR forestr* OR rubber plantation* OR plantation* OR palm oil OR fisher* OR fishing OR manufacturing OR retail* OR construction OR agricultur* OR farm* OR brick kiln* OR domestic work*)adj3(anxiety OR depress* OR psychiatric OR bipolar OR psychos* OR schizophren* OR fear OR guilt OR hostil* OR shame OR suicid*)).mp. |
| 6 | 1 AND (2 OR 3 OR 4 OR 5) |
| **Search concept 2 transnational families of labour migrants** | |
| 7 | exp Child/ OR exp Infant/ OR exp Infant, Newborn/ OR exp Child, Preschool OR exp Adolescent/ OR (child* OR infant OR adolescen* OR young adult* OR teen* OR young person* OR juvenile OR boy OR girl OR youth OR pupil* OR student* OR newborn OR baby).mp. |
| 8 | exp Parents/ OR exp Mothers/ OR exp Fathers/ OR exp Legal Guardians/ OR exp Caregivers/ OR (parent* OR mother OR father OR guardian* OR caregiver*).mp. |
| 9 | (parent* migrat* OR labo?r sending countr* OR transnational famil* OR left ?behind famil* OR Left behind OR Left?behind children).mp. |
| 10 | ((Left behind OR unaccompanied OR abandon* OR left OR leave OR away OR absen* OR separat*) adj3 (child* OR infant OR adolescen* OR young adult OR youth OR girl OR boy OR juvenile OR teen* OR newborn OR baby)).mp. |
| 11 | 1 AND (7 OR 8 OR 9 OR 10) |
| **Search concept 3 Asia-Pacific Region** | |
| 12 | (Asia OR Southeast Asia OR East Asia OR South Asia OR Arab* OR Gulf* OR Afghanistan OR Bahrain OR Bangladesh OR Bhutan OR Brunei Darussalam OR Brunei OR Cambodia OR China OR Chinese OR Cyprus OR Korea* OR Fiji OR India OR Indonesia OR Iran OR Iraq OR Japan* OR Jordan OR Kazakhstan OR Kiribati OR Kuwait OR Kyrgyzstan OR Lao OR Lebanon OR Malaysia OR Maldives OR Marshall Islands OR Micronesia OR Mongolia OR Myanmar OR Nauru OR Nepal OR Oman OR Pakistan OR Palau OR Papua New Guinea OR Philippines OR Filipino OR Qatar OR Samoa OR Saudi Arabia OR Singapore OR Solomon Islands OR Sri Lanka OR Syria* OR Tajikistan OR Taiwan* OR Thailand OR Timor-Leste OR Tonga OR Turkey OR Turkmenistan OR Tuvalu OR United Arab Emirates OR Emirates OR Uzbekistan OR Vanuatu OR Viet Nam OR Vietnam* OR Yemen).mp. |
| **Combine search concepts and limit date range to 2013 onwards** | |
| 13 | (6 OR 11) AND 12 |
| 14 | limit 13 to (yr="2013 -Current") |

**Appendix 3 List of countries and territories in the Asia-Pacific region defined in this review**

This review defines the Asia-Pacific region based on the United Nations regional classification, which includes 55 countries and territories. In addition, Taiwan was included due to its geographic proximity, economic integration with the region, and the presence of relevant research data.

**Countries and territories included**:

Afghanistan, Bahrain, Bangladesh, Bhutan, Brunei Darussalam, Cambodia, China, Cyprus, Democratic People's Republic of Korea, Fiji, India, Indonesia, Iran (Islamic Republic of), Iraq, Japan, Jordon, Kazakhstan, Kiribati, Kuwait, Kyrgyzstan, Lao People's Democratic Republic, Lebanon, Malaysia, Maldives, Marshall Islands, Micronesia (Federated States of), Mongolia, Myanmar, Nauru, Nepal, Oman, Pakistan, Palau, Papua New Guinea, Philippines, Qatar, Republic of Korea, Samoa, Saudi Arabia, Singapore, Solomon Islands, Sri Lanka, Syrian Arab Republic, Tajikistan, Thailand, Timor-Leste, Tonga, Turkey, Turkmenistan, Tuvalu, United Arab Emirates, Uzbekistan, Vanuatu, Vietnam, Yemen, Taiwan
